# Performance Enhancement of a USV INS/CNS/DVL Integration Navigation System Based on an Adaptive Information Sharing Factor Federated Filter

**DOI:** 10.3390/s17020239

**Published:** 2017-02-03

**Authors:** Qiuying Wang, Xufei Cui, Yibing Li, Fang Ye

**Affiliations:** College of Information and Communication Engineering, Harbin Engineering University, Harbin 150001, China; wangqiuying@hrbeu.edu.cn (Q.W.); xufeicui@hrbeu.edu.cn (X.C.); yefang@hrbeu.edu.cn (F.Y.)

**Keywords:** Inertial Navigation System, Celestial Navigation System, Doppler Velocity Log, integrated navigation, federated filter

## Abstract

To improve the ability of autonomous navigation for Unmanned Surface Vehicles (USVs), multi-sensor integrated navigation based on Inertial Navigation System (INS), Celestial Navigation System (CNS) and Doppler Velocity Log (DVL) is proposed. The CNS position and the DVL velocity are introduced as the reference information to correct the INS divergence error. The autonomy of the integrated system based on INS/CNS/DVL is much better compared with the integration based on INS/GNSS alone. However, the accuracy of DVL velocity and CNS position are decreased by the measurement noise of DVL and bad weather, respectively. Hence, the INS divergence error cannot be estimated and corrected by the reference information. To resolve the problem, the Adaptive Information Sharing Factor Federated Filter (AISFF) is introduced to fuse data. The information sharing factor of the Federated Filter is adaptively adjusted to maintaining multiple component solutions usable as back-ups, which can improve the reliability of overall system. The effectiveness of this approach is demonstrated by simulation and experiment, the results show that for the INS/CNS/DVL integrated system, when the DVL velocity accuracy is decreased and the CNS cannot work under bad weather conditions, the INS/CNS/DVL integrated system can operate stably based on the AISFF method.

## 1. Introduction

An unmanned Surface Vehicle (USV) is self-contained unmanned untethered vessel that can transit on the surface of the water autonomously or by remote control [1,2,3]. The navigation system, which can provide the attitude, velocity and position information for the vehicle continuously, is one of the most important USV subsystems. Many kinds of navigation system, for example, Global Navigation Satellite System (GNSS), Inertial Navigation System (INS), Doppler Velocity System (DVL), are installed on the USVs at present [4,5,6,7,8].

INS can provide the navigation information for USV without external sensors, which cannot be influenced by external factors. However, the navigation information provided by INS can only maintain high precision for short time limited by the method [9,10]. Therefore, the INS navigation error will accumulate over time and diverge after a long duration [11,12,13].

A method to mitigate and limit the error is by updating the inertial system periodically or continuously with external aid. Hence, the navigation accuracy can be greatly improved by external measurements when an external aid is available aboard the vehicle. GNSS is a prevalent choice for INS augmentation. Reference [14] introduced the principle of INS/GNSS integrated systems in detail; the method to obtain the navigation information by INS/GNSS has been researched comprehensively and thoroughly based on the principles [15,16]. Although the navigation error of INS is corrected periodically and continuously by the integrated navigation methods, these methods cannot work under GNSS signal outage conditions. For example, when the number of satellites is less in some pockets of the Earth, or the satellites are damaged, the integrated system INS/GNSS cannot work anymore. Therefore, the navigation technology without GNSS for short time (from several minutes to ten minutes) is a research focus in the navigation field [17,18]. Qin et al. [13] proposed a Self-Constructive Adaptive Network-based Fuzzy Inference System (SCANFIS) combined with the Extended Kalman Filter (EKF) for MEMS-INS error modeling and prediction to improve the positioning accuracy when a GNSS signal is blocked. Noureldin [19] proposed an approach to enhance the positioning accuracy during GNSS outages by nonlinear modeling of INS position errors at the information fusion level using Neuro-Fuzzy (NF) modules, which are augmented in the INS/GNSS integration system based on a Kalman Filter. However, the integrated navigation methods above are designed for the GNSS signal outages of short duration, and the integrated navigation based on these methods cannot work when the GNSS signal is blocked for a long time (several hours or some days for USVs).

CNS provides the position information of a vehicle by taking the stars as the beacon, which is immune to electromagnetic interference and has high navigation accuracy with no accumulative error. The positioning accuracy of CNS can be better than 10 m in theory [20]. At present CNS is applied in the aerospace field, and it has become a popular application in the field of USV navigation in recent years. Hence, CNS can be used to correct the INS divergence navigation error in some applications [21,22,23,24]. Compared with GNSS, CNS, which is introduced to correct the error of INS, can navigate by taking stars as objects observed, and stars exist everywhere and cannot be damaged by human behavior. Star sensors are the most cutting-edge CNS technique [25,26,27,28,29]. A star sensor is an electronic camera connected to a microcomputer. However, when the star sensor is applied to surface ships, the navigation process is easily affected by bad weather and other factors. Hence, the navigation information provided by the star sensor cannot be obtained accurately, which can lead to failure. In this case, the accuracy of INS/CNS integrated navigation is reduced. However, few papers can be found about resolving the problem that the reduction of integrated navigation accuracy caused by the star sensor. Therefore, there is a further research needed to keep the integrated navigation system working stable when the star sensor cannot work. 

DVL, which is designed based on the Doppler Effect, can provide the USV’s velocity when the vehicle is in sufficiently shallow water to provide a bottom lock. Hence, the divergence navigation error of the INS can be corrected by the velocity information from DVL when the vehicle is in shallow water [7,30,31,32,33]. The principle of the integrated method of INS/DVL is estimating INS error by a Kalman Filter, and the observation is the difference between the INS velocity and the DVL velocity [34,35,36]. The main purpose of method is correction of the growth of INS unbounded errors using the velocity information from DVL. It is assumed that the velocity accuracy of DVL is better than that of INS. Therefore, the DVL velocity is used as the reference information to correct the INS navigation error. However, the integration accuracy based on DVL/INS can be influenced by some resources, for example, the rotation matrix between DVL and INS, the DVL measurement error and so forth. Troni [37] proposed the development of new methods for the problem of in situ calibration of the alignment rotation matrix between Doppler sonar velocity sensors and attitude sensors arising in the navigation of underwater vehicles and performed a comparative performance evaluation, using laboratory and at-sea field data. References [38,39] show the methods of calibrating the DVL measurement error, which is caused by the DVL itself, ocean currents and so on. However, not all of the DVL measurement errors can be estimated and corrected. There are still residual DVL measurement errors which cannot be corrected. Hence, the accuracy of the INS/DVL integrated navigation system is reduced by the DVL measurement residual errors. Therefore, how to estimate and correct the INS unbounded position error should be researched.

According to the statement above, it can be seen that both the CNS and DVL have shortcomings as the aiding references. Therefore, the DVL velocity and the CNS position serve as the reference information to correct the INS navigation error at the same time, and the integrated navigation approach based on INS/CNS/DVL using the federated filter is proposed in this paper. 

The federated filter, proposed by Carlson [40], is a two-stage data processing technique. Sensor-related filters are subsequently processed and combined by a larger master filter. The advantage of this filter is that a subsystem which works at a fault state can be detected, and then it can make other subsystems operate normally [41,42,43]. Hence, the Federated Filter can be used as the data fusion method for an INS/CNS/DVL integrated navigation system. When the CNS cannot work in bad weather or the accuracy of the DVL is not good enough for the integration navigation system, the faults of the navigation subsystem (CNS or DVL) of the integration navigation system can be detected and separated by the Federated Filter, and the influence of the reference information accuracy obtained from CNS or DVL in the integration system can be avoided. In addition, an important parameter of the Federated Filter is the Information Sharing Factor (ISF), which can judge the “contribution” of each sub filters, and the performance of the Federated Filter is determined by ISF. Hence, it is of importance to set the value of ISF. In Carlson’s original Federated Filter the information is shared equally between filters, so the ISF for each filter is the same (e.g., for three filters, ISF = 1/3). More recent works adaptively change the ISF based on the eigenvalues of the covariance matrix [44] or the trace of the covariance matrix [45]. In this paper, we adjust the ISF based on the difference between the expected covariance (which can be calculated directly) and the observed covariance over a window of M samples. This allows our federated filter to identify unexpected degradation in measurement quality. Therefore, an approach of setting ISF adaptively based on the innovation covariance is proposed, which is purposely designed for the INS/CNS/DVL integrated system in this paper. 

The principle and the error analysis of INS, CNS and DVL are described in Section 2. The error analysis result based on the principle and the experiment shows the reason of introducing the INS/CNS/DVL as the integrated navigation method. Section 3 presents the proposed method of the Federated Filter based on the adaptive ISF. The principle of the integration navigation system of INS/CNS/DVL based on ISF Federated Filter is shown in Section 4. Section 5 presents the simulation and experimental results, followed by the conclusions for Section 3 and Section 4. Section 6 summarizes the whole paper.

## 2. Principle of the Integration Subsystem and Error Analysis

### 2.1. Principle of INS and System Error Model

#### 2.1.1. Principle of INS

**Figure 1:**
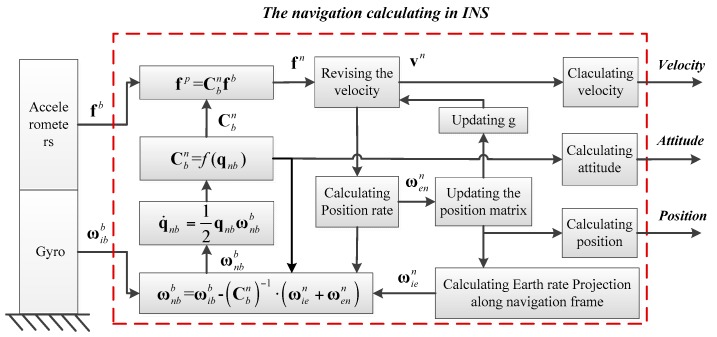
Principle of INS.

Gyros and accelerometers are the main Inertial Measurement Units (IMUs) of INS, and the vehicle’s navigation information is obtained by the calculation process based on the IMU measurement. The principle of INS is shown in Figure 1 [46,47,48].

In the Principle of INS, the script *i* denotes the Earth-Centered Inertial frame (ECI), *n* denotes the navigation frame (East-North-Up, ENU), e denotes the Earth-Centered Earth-Fixed frame (ECEF), and *b* denotes the body-fixed frame. $C_{b}^{n}$ denotes a transformation matrix from *b* frame to *n* frame. **f**^*b*^ denotes the accelerometer output, $\omega_{ib}^{b}$ denotes the gyro output. $\omega_{xy}^{z}$ (*x* = *i*,*e*,n; *y* = *e*,*n*,*b*; *z* = *n*,*b*) denotes the rotation rate along *z* between *x* and *y*. **q***_nb_* denotes the rotating quaternion between *b* and *n*. 

Based on the principle of INS, the position, velocity, and attitude equations for INS are expressed in (1). Compared with the plane and spaceflight system, the ship’s altitude is changed less than 100 m. Therefore, in order to simplify the problem, the navigation information related with the altitude in the INS error equation is ignored because the research topic of this paper is USVs. The current position, velocity, and attitude can be obtained by integrating these equations with the angular rate and the acceleration data from IMU in real time:

(1) $$\{\begin{array}{l} \dot{P}=v^{n}R\\ {\dot{v}}^{n}=C_{b}^{n}f^{b}-\left(2\omega_{ie}^{n}+\omega_{en}^{n}\right)\times v^{n}+g^{n}\\ \dot{q}=q⊗\omega_{nb}^{b}/2 \end{array}$$

where **P** = [*ϕ λ*]^*T*^ and **R** = *diag*([1/*R_m_* 1/(*R_t_*cos*ϕ*)]. *v_x_* and *v_y_* denote the east and north velocity along *n* frame calculated by INS. *ϕ* and *λ* denote the latitude and the longitude calculated by INS. **g**^*n*^ denotes gravity along *n* frame. *R_m_* denotes the semi major axis of the Earth, *R_t_* denotes the semi minor axis of the Earth.

#### 2.1.2. Error Analysis for INS

Based on the principle of INS, the velocity error, misalignment angles and the position error calculated by INS are the state variables, and the INS error model is shown as follows:

(2) $$\dot{X}=A_{0}X$$

where:

(3) $$X={\left[\begin{array}{ccccc} \delta P^{T} & \delta v^{T} & \phi^{T} & \nabla^{T} & \varepsilon^{T} \end{array}\right]}^{T}$$

(4) $$A_{0}=\left[\begin{array}{ccccc} A_{\mathrm{PP}} & A_{\mathrm{Pv}} & O_{3\times 3} & O_{3\times 3} & O_{3\times 3}\\ A_{\mathrm{vp}} & A_{\mathrm{vv}} & A_{v\phi } & A_{v\nabla } & O_{3\times 3}\\ A_{\phi P} & A_{\phi v} & A_{\phi \phi } & O_{3\times 3} & A_{\phi \varepsilon }\\ O_{3\times 3} & O_{3\times 3} & O_{3\times 3} & O_{3\times 3} & O_{3\times 3}\\ O_{3\times 3} & O_{3\times 3} & O_{3\times 3} & O_{3\times 3} & O_{3\times 3} \end{array}\right]$$

where *δ***P** = [*δϕ δλ*]^*T*^ denotes the position error; *δ***v**[*δv_x_ δv_y_*]^*T*^ denotes the velocity error; φ = [*φ_x_*,*φ_v_*,*φ_z_*]^*T*^ denotes pitch, roll, yaw misalignment angles, respectively, and the initial value of the misalignment angles is stationary, but considering the influence of IMU errors, the misalignment angles varies with time, oscillating during the navigation process 14; ∇ = [∇_*x*_,∇_*v*_,∇_*z*_]^*T*^ denotes accelerometers bias, **ε** = [*ε_x_*,*ε_v_*,*ε_z_*]^*T*^ denotes the gyro drift. **O**_3×3_ denotes the matrix with three rows and three columns, and all of the elements of the matrix are zero; **A***ij* (*i* = **P**,**v**,φ, *j* = **P**,**v**,φ,∇,**ε**) denotes the transformation matrix between *j* and *i*.

(5) $$A_{\mathrm{PP}}=\left[\begin{array}{cc} 0 & 0\\ v_{x}\sec\varphi \tan\varphi /R & 0 \end{array}\right],\;A_{\mathrm{Pv}}=\left[\begin{array}{cc} 0 & 1/R\\ \sec\varphi /R & 0 \end{array}\right],\;A_{\mathrm{vP}}=\left[\begin{array}{cc} 2\omega_{iey}^{n}v_{y}+v_{x}v_{y}\sec^{2}\varphi /R & 0\\ -\left(2\omega_{iey}^{n}v_{x}+v_{x}^{2}\sec^{2}\varphi /R\right) & 0 \end{array}\right],$$

(6) $$A_{\mathrm{vv}}=\left[\begin{array}{cc} v_{y}\tan\varphi /R & 2\omega_{iez}^{n}+v_{x}\tan\varphi /R\\ -\left(2\omega_{iez}^{n}+v_{x}\tan\varphi /R\right) & 0 \end{array}\right],\;A_{\phi P}=\left[\begin{array}{cc} 0 & 0\\ -\omega_{iez}^{n} & 0\\ \omega_{iey}^{n}+v_{x}\sec^{2}\varphi /R & 0 \end{array}\right],\;A_{\phi v}=\left[\begin{array}{cc} 0 & -1/R\\ 1/R & 0\\ \tan\varphi /R & 0 \end{array}\right],$$

(7) $$A_{\phi \phi }=\left[\begin{array}{ccc} 0 & \omega_{iez}^{n}+v_{x}\tan\varphi /R & -\left(\omega_{iey}^{n}+v_{x}/R\right)\\ -\left(\omega_{iez}^{n}+v_{x}\tan\varphi /R\right) & 0 & -v_{y}/R\\ \left(\omega_{iey}^{n}+v_{x}/R\right) & v_{y}/R & 0 \end{array}\right],\;A_{v\phi }=\left[f^{n}\times \right],\;A_{v\nabla }=C_{b}^{n},A_{\phi \varepsilon }=C_{b}^{n}$$

where *R* ≈ *R_m_* denotes the radius of the Earth, $\omega_{iey}^{n}=\omega_{ie}\cos\varphi$, $\omega_{iez}^{n}=\omega_{ie}\sin\varphi$ denotes the component of angular velocity of earth rotation along the o*y* axis, o*z* axis of the navigation frame respectively.

### 2.2. Principle of CNS and Error Analysis

#### 2.2.1. Principle of CNS

**Figure 2:**
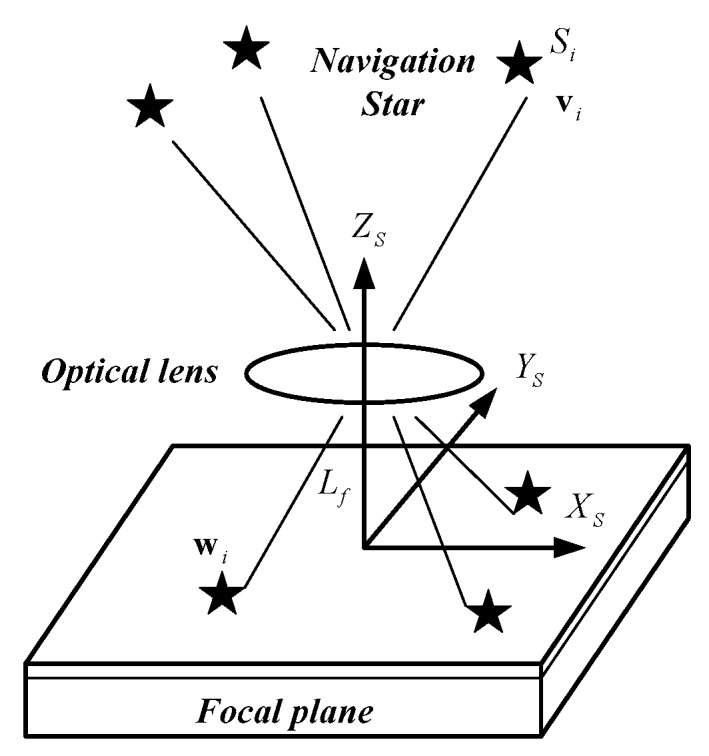
Principle of the star sensor.

A star sensor is an electronic camera connected to a microcomputer. Stars can be located and identified by using a sensed image of the sky. The orientation of the vehicle can be determined by the observation [49]. As shown in Figure 2, navigation star *S_i_* with direction vector **v**_*i*_ in the celestial coordinate system can be detected by the star sensor, whereas the vector of its image can be expressed as **w**_*i*_ in the star sensor coordinate system.

The position of the principal point of the star sensor on the image is defined as (*x*_0_,*y*_0_). The position of the image point of navigation star *S_i_* on the image plane is (*x_i_*,*y_i_*). The focal length of the star sensor is *L_f_*. Vector **w**_*i*_ can be expressed using Equation (3) [48,49]:

(8) $$w_{i}=\frac{1}{\sqrt{{\left(x_{i}-x_{0}\right)}^{2}+{\left(y_{i}-y_{0}\right)}^{2}+L_{f}^{2}}}\left[\begin{array}{c} \left(x_{i}-x_{0}\right)\\ \left(y_{i}-y_{0}\right)\\ L_{f} \end{array}\right]$$

where **w**_*i*_ is the vector projection of the image point of navigation star *S_i_* on the image plane.

Equation (3) elucidates the relationship between **w**_*i*_ and **v**_*i*_ under ideal conditions, where **A** is the attitude matrix of the star sensor:

(9) $$w_{i}=Av_{i}$$

In theory, when the number of navigation stars is more than two, the attitude matrix can be solved using a math algorithm (for example, QUEST algorithm, SVD algorithm and so on). Thus, the optimal attitude matrix in the inertial space of the star sensor can be calculated. Based on the principle, an autonomous star sensor is an avionics instrument adopted to provide the absolute 3-axis attitude of a spacecraft utilizing star observations. Therefore, the attitude accuracy of an autonomous star sensor is the main factor.

#### 2.2.2. Error Analysis for CNS

Compared with the INS error, the star sensor accuracy is much better than that of INS. Therefore, the INS navigation error can be corrected by the star sensor. However, the average number of stars in the field of view (FOV) and the brightness of the stars are very important to the accuracy and the percentage of the sky where the star sensor operates. Reference [49] shows the average number of stars in the FOV is:

(10) $$N_{FOV}=6.57\cdot e^{1.08M}\cdot \frac{1-\cos\left(A/2\right)}{2}$$

where *N_FOV_* is the total number of stars on the FOV. *M* is the visual magnitude. It is assumed that the FOV is circular and is *A* degrees wide.

Therefore, the star sensor attitude accuracy is reduced with the reduction of the average number of stars in the FOV, which can be affected by weather factors (such as clouds), then the navigation information accuracy of the star sensor is reduced with it. 

**Figure 3:**
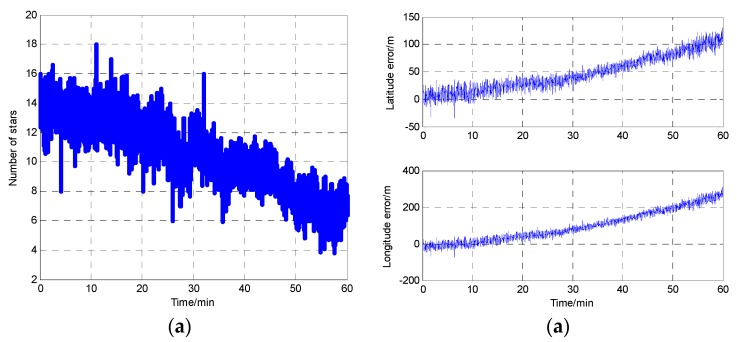
Star sensor navigation result in the poor weather condition. (**a**) The number of stars available; (**b**) star sensor based CNS positioning error.

In order to prove the viewpoint that the star sensor accuracy can be influenced by the number of stars in the FOV, an experiment is performed. In the experiment, the star sensor is installed on the ground outside. The number of stars in FOV is collected by computer. During the experiment process, sometimes there are clouds in the sky. Hence, the number of stars in the FOV is changed. In addition, the position (latitude and longitude) of the experiment location are known before the experiment, so the difference between the known position information and the star sensor position information can be used as the judging standard of star sensor accuracy. Figure 3 shows the experiment result within 60 min under the bad weather. Figure 3a is the number of available stars during this time period, and the positioning error provided by the star sensor during this process is shown in Figure 3b. Both figures take time as the abscissa, and the results in these two figures are in the same time. Therefore, Figure 3 shows the star sensor position accuracy is changed with the number of available stars.

From Figure 3, due to the clouds and other external factors, the number of available stars decreases gradually and the star sensor positioning errors increase with it. Therefore, the positioning accuracy of the star sensor will decrease when the number of stars in the FOV is decreased under poor weather conditions. 

According to the CNS principle, the CNS position error depends on the number of available stars. Therefore, the measurement covariance for the CNS is designed as a function of the number of stars observed. Based on the curves in Figure 3, the relationship between the number of star observed and the CNS position error is designed by fitting data (using the “*polyfit*” function in Matlab), and it is:

(11) $$y(t)=0.0636x^{3}+1.4732x^{2}-78.2011x+632.6667$$

where *x* is the number of star observed in CNS; *y* is the position error, whose unit is meters.

### 2.3. Principle of DVL and Error Analysis

#### 2.3.1. Principle of DVL

**Figure 4:**
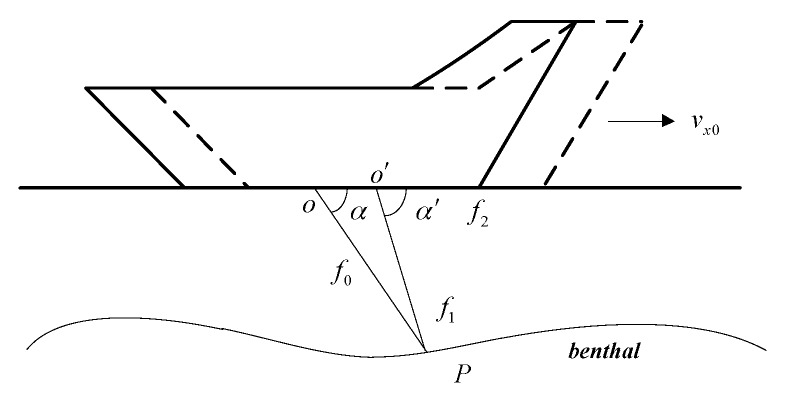
The principle of DVL.

The vehicle’s velocity can be measured by the Doppler Velocity Log (DVL), which is designed by the Doppler Effect. The principle of DVL is shown in Figure 4.

Here *f*_0_ denotes the frequency of the DVL emission signal. *f*_1_ denotes the frequency of the signal at point P. *f*_2_ denotes the frequency of the reflected signal. *c*_0_ is the velocity of the signal, *v_x_*_0_ denotes the vehicle’s velocity along its heading. The wave source moves from point *O* to *O*’ when the reflected signal is received by DVL. 

#### 2.3.2. Error Analysis for DVL

**Figure 5:**
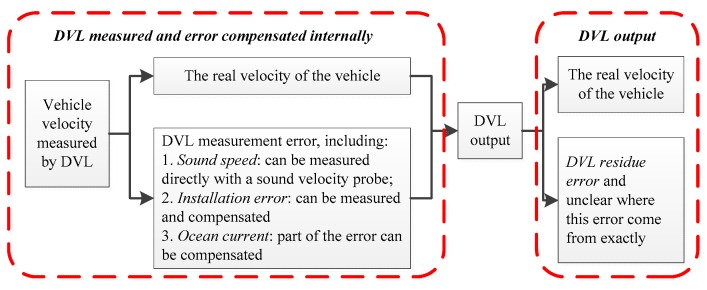
Relationship between DVL measurement error and residue error.

Sound speed, installation error and ocean currents are the main DVL error factors, and most of the DVL errors caused by these factors can be calculated and compensated before DVL velocity output. However, not all DVL velocity errors can be compensated, which means that there is a residual error as the component of DVL velocity output and influence the DVL accuracy [36,37,50,51,52]. It means that the components of DVL output includes the vehicle’s real velocity and the DVL residue error, and the DVL residue error is unclear where it comes from. Therefore, the DVL residue error is main error, which is the most concern in this paper. The relationship described above is shown in Figure 5.

For the integrated navigation INS/DVL, the precondition of using DVL as the reference system is that the accuracy and stability of DVL is better than INS, and this condition can be satisfied in most cases although the DVL velocity is not absolutely accurate. The integrated navigation accuracy can be improved if the DVL residue error is modeled and compensated. This means that the form of the DVL velocity residue error is more concerned, and the source of this error is not of concern in this paper. Hence, how to determine the mathematical form of the DVL velocity residue error and avoid the influence of the DVL residue error on the integrated navigation method is the main problem. 

In order to model the DVL residual error, one group of DVL at-sea field data is used, and the mathematical form of the DVL residual error is derived by the measured data. In the process of the experiment, the vehicle-installed DVL is navigating on the sea for 60 h, and the navigation trajectory is along the Chinese coastline. DVL is used as the independent system in the experiment, and part of the DVL error is estimated and adjusted before output. When the vehicle is navigating, the DVL velocity is collected and saved by the computer. In addition, there is another system to provide the high velocity information (provided by PHINS and produced by IXSEA, France), which is also collected and saved by the computer at the same time.

**Figure 6:**
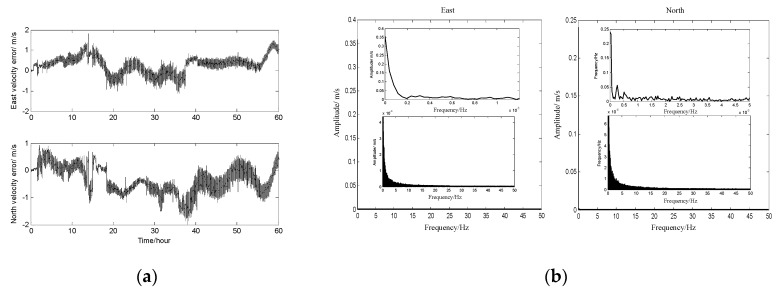
DVL velocity errors in both time and frequency domain. (**a**) DVL velocity error; (**b**) amplitude-frequency along east and north.

The DVL velocity residue error, which is the difference between the DVL velocity and the reference velocity, is shown in Figure 6a. From the curves, it can be seen that the residual velocity error is full of noise and continuous variation, and the form of the DVL velocity error cannot be obtained. In this paper, the form of the DVL velocity error is more of concern than the error source of the DVL velocity residue error. Therefore, the analyzed method by frequency is carried out to confirm. Figure 6b is the amplitude-frequency curves of the DVL velocity error along east and north during 60 h, and these frequency curves are obtained by Fourier transformation (the *fft* function in Matlab) with the DVL velocity error in Figure 6a. The meaning of the amplitude-frequency curve is the velocity component with different frequency, and the two small curves in figures are the enlarged drawing.

Based on the curves in Figure 6b, it can be obtained that the DVL velocity residue error at 0 Hz is 0.455 m/s (the resultant velocity based on the east and north velocity error). The experiment has the velocity on the order of 8 m/s and the velocity error magnitude is 0.455 m/s, that is a 5% error. Because the vehicle is navigating along the Chinese coastline for 60 h during the experiment, and most of the DVL velocity error, which is caused by the sound speed, and installation errors, is compensated by the DVL system itself before the measurement velocity output. It is concluded that most of the DVL residual constant error is caused by ocean current. In addition, the DVL velocity error at 0.1~50 Hz is less than 0.006 m/s, and the amplitude of the velocity decreased gradually with the increased frequency. It is concluded that the residual error with different frequency is caused by the compensation errors (sound errors, installation errors and so on), which means that not all of the DVL error can be estimated and compensated absolutely. 

Therefore, the constant velocity error is the main factor of the DVL velocity error. The measurement noise with different frequency, whose amplitude is much weaker than that of the constant error, is another component of the DVL velocity error. Hence, it is assumed that the DVL error is constant in a short navigation time. The DVL error model is established as follows:

(12) $$\delta V^{b}=\Delta V^{b}+u_{0}$$

where *δ***V**^*b*^ is the DVL residue error; Δ**V**^*b*^ is the constant error; **u**_0_ is the measurement noise.

Based on the error analysis above, it can be obtained that the INS, which is an autonomous navigation system with rich navigation information, can be used as the main navigation system for the integrated navigation system, and the INS navigation error can be corrected by the reference information from the other system. Hence, the corrected navigation information of INS is the final navigation information of the integrated navigation system; the position information with high precision from CNS can be used as the reference information to correct the INS error during the normal operation of the CNS, working at night in most cases; the velocity information from DVL can be used to correct the INS error continuously, but the premise of using DVL as the reference information is by inhibiting the influence of DVL velocity error on the integrated navigation system.

Therefore, the challenges of using CNS and DVL as the reference systems to correct the INS navigation error are summarized as follows: (1) how to ensure the stability of the multi-sensor integrated navigation system based on INS/CNS/DVL when the CNS cannot work under bad weather conditions; (2) how to estimate and correct the DVL constant error and measurement noise, which can lead to the fact the DVL velocity that cannot be used as the reference information and decreases the accuracy of the multi-sensor integrated navigation system. All of the questions above are the main problems that need to be solved in next sections.

## 3. Federated Filter Based on the Adaptive Information Sharing Factor

### 3.1. The Principle of Federated Filter

There are two data fusion methods introduced in the multi-sensor integrated navigation system: one is the centralized Kalman Filter. The INS position error and the INS velocity error serve as the observed variables for the centralized Kalman Filter. The INS position error is the difference between the INS position and the CNS position, and the INS velocity error is the difference between the INS velocity and the DVL velocity. It is assumed that both the DVL velocity and the CNS position accuracy are better than INS. Therefore, all of the INS navigation error in Equation (2) can be estimated and compensated to improve the INS accuracy. 

Compared with taking the position error or velocity error as the only observable, the advantage of using the centralized Kalman Filter is that the observability of the state variables is improved. The limitations of centralized Kalman Filter methods are that the navigation systems can be illustrated when the system is applied. For example, when the CNS is out of order under bad weather conditions, the position accuracy of CNS is decreased, and the difference between the INS position and CNS position is not the INS position error. Similarly, when the DVL velocity accuracy is influenced by other factors, the difference between the INS velocity and DVL velocity is not the INS error. Based on the principle of the centralized Kalman Filter, once the accuracy of one of the observations decreased, the INS error cannot be estimated exactly. Hence, the stability of the navigation information based on the centralized Kalman Filter is decreased when the observed variables fail due to the DVL error or the CNS is out of order. 

**Figure 7:**
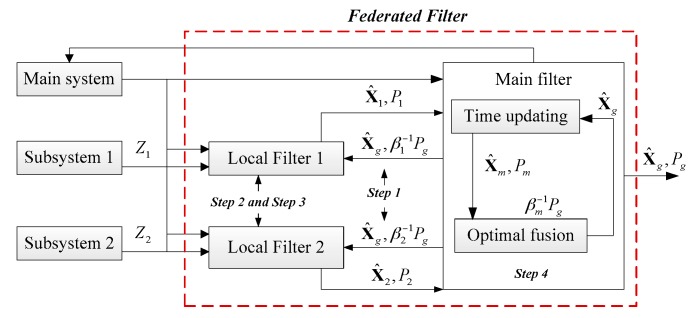
Principle of the Federated Filter.

The other one is the Federated Filter. The Federated Filter is widely for its features of flexible design, good real-time performance and fault tolerance [53]. In concept, the Federated Filter is a two-stage data processing technique in which the outputs of local, sensor-related filters are subsequently processed and combined by a larger master filter, as is illustrated in Figure 7. 

As suggested in Figure 7, the Federated Filter is a dispersed filter in essence, and each local filter is dedicated to a separate sensor subsystem. One or more local filters may also use data from a common reference system. In addition, the initial condition, the noise information and the state variable of each subsystem is updated by the main filter dynamically. Hence, the subsystem information distribution scheme is updated by the updating information of $\beta_{i}^{-1}P_{g}$. For example, when the information accuracy of one of the integrated navigation subsystems is decreased, the mean squared error matrix of subsystem *P_i_* should be adjusted and increased, which means that the estimation error of the subsystem has a big error. Therefore, the mean squared error matrix of subsystem is updated by $P_{i}=\beta_{i}^{-1}P_{g}$.

According to the principle and the equations of Federated Filter, it seems that local covariance is obtained from global covariance, which seems to imply a feedback flow (sending back the fusion result to the subsystem). Take the local filter 1 as an example, and the local covariance *P*_1_ is updated by the equation as [53]:

(13) $$P_{1}^{-1}=\beta_{1}\left[P_{1k}^{-1}{\left(I-K_{1}H_{1}\right)}^{-1}+P_{2k}^{-1}{\left(I-K_{2}H_{2}\right)}^{-1}\right]$$

If the local covariance *P*_1_ is computed independently, it can be obtained that:

(14) $$P_{1}^{-1}=P_{1k}^{-1}{\left(I-K_{1}H_{1}\right)}^{-1}$$

If the local covariance *P*_1_ is computed by global output, it can be obtained that:

(15) $$P_{1}^{-1}=\left[P_{1k}^{-1}{\left(I-K_{1}H_{1}\right)}^{-1}+P_{2k}^{-1}{\left(I-K_{2}H_{2}\right)}^{-1}\right]{\left(I-K_{1}H_{1}\right)}^{-1}$$

Based on the Equations (8)–(10), it seems that if *P*_1_ is computed independently, *P*_1_ is only influenced by the local filter 1 itself. It means that when the information accuracy of one of the integrated navigation subsystems is decreased, *P_i_* should not be adjusted, which means that the estimating error of the subsystem is a big error. If *P*_1_ is computed by global output, it can be adjusted by two local filters. However, the adjustment process is changeless, and the system fault cannot be checked and separated by the Federated Filter.

Therefore, the information sharing factor of *β_i_* is a new parameter, and the proportion of every subsystem in the main filter is adjusted by *β_i_*. It means that the proportion of every subsystem in the main filter increased with the increase of *β_i_*; otherwise the proportion is decreased. Therefore, the information sharing factor of *β_i_* in the breakdown subsystem should be adjusted lower, so that the influence of the breakdown subsystem to the main system is decreased and avoided and the accuracy of the whole filter is stable. It is obtained that setting the suitable value of *β_i_* according to the statement of subsystem is of importance.

According to adjusting process, the system fault can be checked and separated by the Federated Filter. The remaining subsystems, which are operating smoothly, are reconstructed to obtain the integrated navigation information by the Federated Filter. Therefore, the requirement of ensuring the stability and high precision of the navigation information for the multi-sensor integrated navigation system based on the INS/CNS/DVL can be met by introducing the Federated Filter as the data fusion method. Based on the principle of the federated filtering, how to set the information sharing factor *β_i_* is the key to improve the fault-tolerance performance of Federated Filter.

### 3.2. Adaptive Information Sharing Factor for Federated Filter

The Information Sharing Factor (ISF) *β_ik_*, which is the “contribution” of the local filter to the main filter, is an important factor for the Federated Filter. Especially for the multi-sensor integrated navigation system based on INS/CNS/DVL, the reference information accuracy from DVL and CNS is the main factor and can influence the navigation accuracy. Hence, the DVL and CNS information accuracy must be shown by ISF, which means that the ISF must be adjusted adaptively. The principle of setting the ISF of *β_ik_* is shown as follows.

The innovation covariance of the Kalman Filter (local filter 1 and local filter 2 in Figure 7) is:

(16) $$C_{ik}=E[\eta_{ik}\eta_{ik}^{T}]=H_{ik}P_{ik}^{-}H_{ik}^{T}+R_{ik}$$

where *i* = 1,2 denotes the Kalman Filter 1 and Kalman Filter 2 in Figure 7. $\eta_{ik}=Z_{ik}-H_{ik}{\hat{X}}_{ik}$, $P_{ik}^{-}$ and **R**_*ik*_ are innovations, a predicted of the Kalman Filter respectively. **C**_*ik*_ is referred to as the calculated innovation covariance in this paper. 

In general, the innovation of the filter is easily affected by unaccounted errors, such as an unknown fault bias, an unmodeled dynamic or unknown initial condition. In addition, the innovation covariance shows the effect of any unaccounted errors, since they are directly involved in the calculations of the innovation.

For example, if we know an exact dynamic equation, the innovation covariance is equal to **C**_*ik*_, but sometimes, the exact dynamic equation is not available. Then, an estimation error and a predicted error covariance may increase by the effect of the unknown information. Similarly, the exact measurement equation is not available. Then, an innovation covariance **C**_*ik*_ may be increased by the effect of unknown information. In this case, an innovation covariance **C**_*ik*_ is increased by an increased measurement covariance **R**_*ik*_. The change of an innovation covariance can be used for an adaptive filter. The increased innovation covariance can be estimated as:

(17) $${\bar{C}}_{ik}=\frac{1}{M-1}\sum_{j=k-M+1}^{k}\eta_{ij}\eta_{ij}^{T}$$

where *M* denotes a window size, which is related to the performance and sampling frequency of every sensors. ${\bar{C}}_{ik}$ is an estimated innovation covariance in this paper.

Estimating the relationship between the innovation covariance **C**_*ik*_ and the estimated innovation covariance ${\bar{C}}_{ik}$:

(18) $$\alpha_{ik}=\left|tr\left(C_{ik}\right)-tr\left({\bar{C}}_{ik}\right)\right|$$

where *tr*(•) denotes the trace of the matrix. *α_ik_* denotes the math relationship between the innovation covariance ${\bar{C}}_{ik}$ and the estimated innovation covariance ${\bar{C}}_{ik}$.

**C**_*ik*_ is the estimated result of observation at current time point, and ${\bar{C}}_{ik}$ is the mean value of observation estimated result in a period of time. Therefore, the factor *α_ik_* shows the stability of the observation estimated result at the current time point. When the measurement noise is known exactly, the numerical value of **C**_*ik*_ has been checked against the value of ${\bar{C}}_{ik}$, and the value of *α_ik_* is approximate to zero; when the measurement noise make a sudden change and the observation accuracy is decreased at time point k, the innovation covariance **C**_*ik*_ at time point k is deviated from the estimated innovation covariance ${\bar{C}}_{ik}$ in a last period of time, and the value of *α_ik_* is increased. Therefore, the value of *α_ik_* has a direct ratio relation with the measurement noise. Hence, the ISF can be designed by *α_ik_*, the calculation method is shown as follows:

(19) $$\beta_{ik}=\frac{\alpha_{ik}^{-1}}{\sum_{i=1}^{2}\alpha_{ik}^{-1}}$$

The relationship between the stability the measurement noise and the ISF concerns into inverse ratio, hence, the form of *α_ik_* in Equation (14) is the inverse matrix. If there is a big difference between the measurement noise at current time point and the setting value, it is obtained that the observation is poor and the “contribution” of this local filter is decreasing to the main filter; conversely, the difference between the measurement noise at current time point and the setting value is small, it is obtained that the observation is good and the “contribution” of this local filter is increasing to the main filter. Thus, the ISF is adjusting adaptively in real time and improving the accuracy and the stability of the Federated Filter. 

In addition, the purpose of introducing the adaptive ISF is to resolve the influence of the observation accuracy on the multi-sensor integrated navigation, and there is no observation for the main filter. Therefore, it is assumed that the ISF for the main filter is *β_m_* = 0.

## 4. The Multi-Sensor Integrated Navigation Method Using the Adaptive ISF Federated Filter

**Figure 8:**
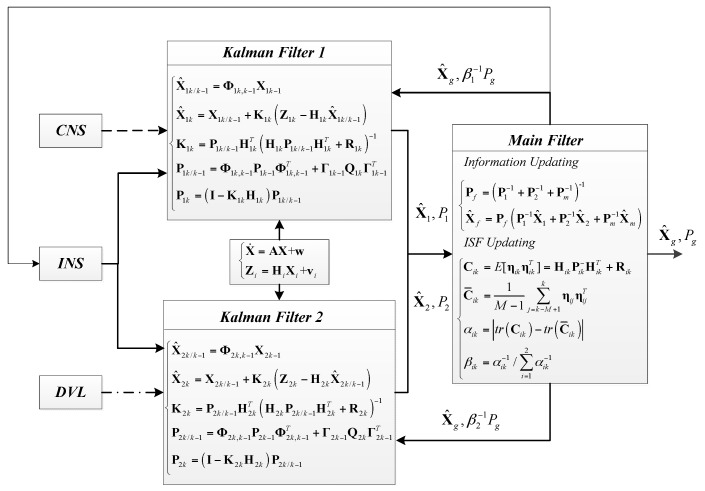
Construction of the multi-sensor integrated navigation method based on the AISFF.

Based on the principle of the sensors in Section 2 and the Adaptive ISF Federated Filter (AISFF) in Section 3, the multi-sensor integrated navigation method for INS/CNS/DVL based on the adaptive ISF Federated Filter is proposed in this section. In this method, INS is introduced as the main system, and CNS and DVL are introduced as the subsystems. Therefore, the CNS position information and the DVL velocity information are used to compensate INS navigation errors. The local filter is the Kalman Filter. The construction of the multi-sensor integrated navigation method based on the adaptive ISF Federated Filter is shown in Figure 8.

The dynamic and the measurement equation for the local filter are:

(20) $$\{\begin{array}{l} \dot{X}(t)=A(t)X(t)+w(t)\\ Z_{i}(t)=H_{i}(t)X(t)+v_{i}(t) \end{array}$$

where **w**(*t*) and **v**_*i*_(*t*) denote the state noise matrix and the measurement noise respectively; **X** denotes the state variable and the form is:

(21) $$X={\left[\begin{array}{llllll} \delta P^{T} & \delta v^{T} & \phi^{T} & \nabla^{T} & \varepsilon^{T} & \delta v_{DVL}^{T} \end{array}\right]}^{T}$$

where *δ***v**_*DVL*_ denotes the DVL velocity constant error, and the form is *δ***v**_*DVL*_ = [*δ***v**_*xDVL*_ *δ***v**_y*DVL*_]^*T*^. *δ***v**_*xDVL*_ and *δ***v**_*yDVL*_ denote the velocity constant error along east and north of the navigation frame.

**A** is the state transition matrix and the form is:

(22) $$A=\left[\begin{array}{cc} A_{0(15\times 15)} & O_{15\times 2}\\ O_{2\times 15} & O_{2\times 2} \end{array}\right]$$

where **A**_0(15×15)_ is the transformation matrix and the form is shown in Equation (2).

**H**_1_ is the measurement matrix of Kalman filter 1 and the form is:

(23) $$H_{1}=\left[\begin{array}{cc} I_{2\times 2} & O_{2\times 13} \end{array}\right]$$

**Z**_1_ is the observations of the measurement equation in Kalman filter 1, which is the difference between the INS position and the CNS position, it is:

(24) $$Z_{1}={\left[\begin{array}{cc} \varphi -\varphi_{CNS} & \lambda -\lambda_{CNS} \end{array}\right]}^{T}$$

where *ϕ* and *ϕ_CNS_* are the latitude information from INS and CNS respectively, *λ* and *λ_CNS_* are the longitude information from INS and CNS, respectively.

**H**_2_ is the measurement matrix of Kalman filter 2 and the form is:

(25) $$H_{2}=\left[\begin{array}{cccc} O_{2\times 2} & I_{2\times 2} & O_{2\times 9} & -I_{2\times 2} \end{array}\right]$$

**Z**_2_ is the observations of the measurement equation of Kalman filter 2, which is the difference between the INS velocity and the DVL velocity, it is:

(26) $$Z_{2}={\left[\begin{array}{cc} v_{x}-v_{xDVL} & v_{y}-v_{yDVL} \end{array}\right]}^{T}$$

where *v_x_* and *v_y_* are the velocity calculated by INS along east and north of the navigation frame. *v_xDVL_* and *v_yDVL_* are the velocity from DVL along east and north of the navigation frame.

In addition, ${\hat{X}}_{f}$ is the state vector for the global solution. ${\hat{X}}_{g}$ is the final estimating result for the AISFF. According to the background of INS/CNC/DVL, it is assumed that ${\hat{X}}_{f}={\hat{X}}_{g}$ [53].

Based on the principle in Figure 8, the observation parameter of Kalman Filter 1 is the difference between the INS position and the star sensor position, and the observation parameter of Kalman Filter 2 is the difference between the INS velocity and the DVL velocity. Then the estimation results (including the INS error and the DVL error) and the mean squared error matrixes **P**_*i*_(*i* = 1,2) of these two filters are the input information of the main filter. Then, the final estimation results, which are the INS error and the DVL error, are obtained by integrating the input information from two Kalman Filters. The estimation results are fed back and compensated to correct the INS navigation information. The better navigation information is obtained. 

At the same time, the ISF is updated by the input information of **P**_1_ and **P**_2_ from two Kalman Filters and the ISF updating method proposed in Section 3. For example, it is assumed that the DVL velocity accuracy is decreased due to the unknown measurement noise at time point k, which means that the setting value of **R***_k_* is inaccurate. Hence, **P**_2_, which is called mean squared error matrixes and reflects the estimation accuracy of Kalman Filter 2, is increased. Then, the innovation covariance **C**_*ik*_ at time point k is increased compared with the average innovation covariance ${\bar{C}}_{ik}$, which is the mean value of the innovation covariance from time point k-M to time point k. The ISF of *β*_2_ is increased based on the calculation method of Equation (14), and the feedback information $\beta_{2}^{-1}P_{g}$ is decreased. Then, the mean squared error matrix of Kalman Filter 2 is adjusted and corrected, and the information updating in the main filter part is also adjusted.

In addition, the DVL velocity constant error *δ***v**_*DVL*_ serves as the state variable. A centralized filter allows observability of the constant errors included in the state vector, and it can be estimated by local filter 1 when the DVL accuracy is not influenced by measured noise. However, if observability is kept with the federated solutions which develop independent computations of state vectors, there is some possible limitation of federated filter in this type of applications, which means that the paradoxical relationship is inevitably produced between the constant error estimating and the DVL measured noise inhibition. The principle of ISF adjusting the Kalman Filter 1 is the same as Kalman Filter 2. The difference between these adjustment processes is that DVL accuracy is decreased by measured noise and CNS accuracy is decreased by the reduction of observation stars. However, the problem is the observation accuracy is decreased for DVL or CNS. Hence, the ISF adjustment principles of these two Kalman Filters are the same. Therefore, the INS navigation error can be estimated and compensated based on the multi-sensor integrated navigation method of INS/CNS/DVL using AISFF. Hence, introducing the DVL velocity constant error as the state variable and taking the ISF adjusting the Kalman Filter are the better method to balance the influence from these two factors and improve the navigation accuracy.

Based on the principle above, it is obtained that setting the value of *M* in Equation (12) is important. How to set the value of *M* is related to the performance and sampling frequency of every sensor. It is assumed that both the data sampling frequency of DVL and Star Sensor is 1 Hz, and comparing the average information ${\bar{C}}_{ik}$ within five minutes to the innovation covariance **C**_*ik*_ at time point *k* The setting value of *M* is 300 (300 = 5 min × 60 s). Hence, the principle of how to set the value of *M* depends on the sensors and environment.

According to the principle of the multi-sensor integrated method based on the adaptive ISF Federated Filter above, it can be seen that, although the computational complexity of the ISF Federated Filter is more intensive than that of a single filter, the reduction of DVL or CNS accuracy can be checked and separated by the ISF Federated Filter. The remaining subsystems, which are operating smoothly, are reconstructed to obtain the integrated navigation information. Therefore, the requirement of ensuring the stability and high precision of the navigation information based on the INS/CNS/DVL can be met by introducing the ISF Federated Filter as the data fusion method.

## 5. Analysis of the Simulation and the Experimental Study

### 5.1. The Simulation Analysis

#### 5.1.1. Simulation Conditions

**Figure 9:**
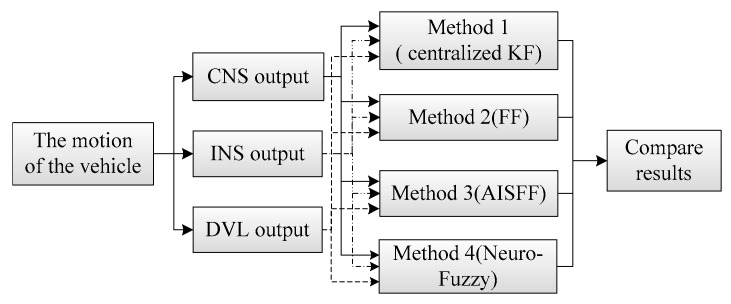
Scheme of the simulation.

Based on the approach, we performed simulations using MATLAB software (MathWorks, Natick, MA, USA) with the simulation conditions as follows, and the scheme of simulation is shown in Figure 9.

##### Motion State of the Vehicle

**Figure 10:**
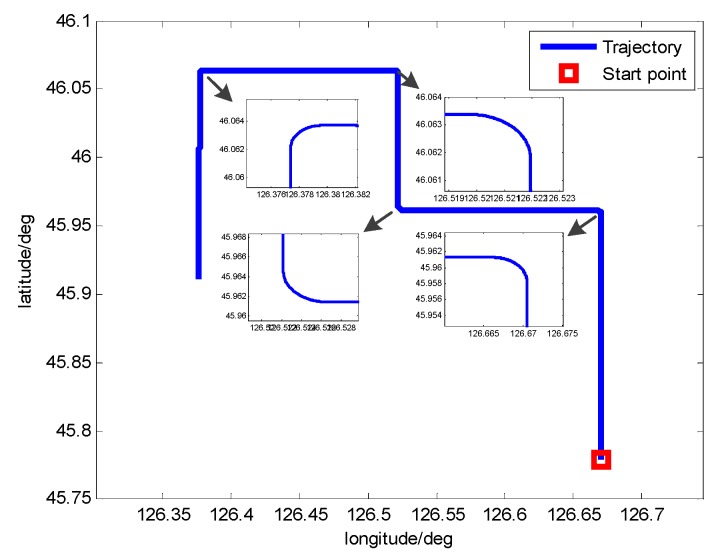
Vehicle’s trajectory of the simulation.

The inertial position of the vehicle is 45.7796° N, 126.6705° E and the initial attitude is 0°, 0°, 30°. The vehicle’s trajectory of the simulation is shown in Table 1 and Figure 10. 

##### IMU, CNS and DVL Error

Gyro error: the constant drift is 0.01°/h, and the measurement noise is Gaussian white noise with 0.001°/h amplitude.

Accelerometer error: the Bias is 10^−5^ g, and the measurement noise is Gaussian white noise with amplitude of 10^−5^ g.

Star sensor error: position error is constant with 10 m. Measurement noise is Gaussian white noise with amplitude of 1 m. The number of star observed is more than 14 in the 0~80 min and 120~180 min; the number decreased to 4, and then increased to 15 linearly during the time 80~120 min, the max position error of CNS 500 m approximately.

DVL error: the constant error is 0.5 m/s. The measurement noise is color noise.

##### The Integrated Navigation Methods and the Parameter Setting

Three integrated navigation methods are introduced in the simulation (shown in Figure 9). One is proposed in this paper, the others are the integrated method with Kalman Filter and the Federated Filter, respectively. Three simulation results are compared in order to prove the validity and superiority of the integrated navigation method proposed in this paper.

*Method 1*:

The centralized Kalman Filter is introduced for the multi-sensor integrated navigation based on INS/CNS/DVL in the method 1. The reason of introducing the centralized Kalman Filter as a comparison is to show that the influence of the observations on the estimation accuracy when one of the observations accuracy is decreased.

The dynamic model for method 1 is the same with Equation (12), and the measurement matrix of the centralized Kalman Filter and the form is:

(27) $$H_{M1}=\left[\begin{array}{llll} I_{2\times 2} & O_{2\times 2} & O_{2\times 9} & O_{2\times 2}\\ O_{2\times 2} & I_{2\times 2} & O_{2\times 2} & -I_{2\times 2} \end{array}\right]$$

Z_*M*1_ is the observations of the measurement equation, which is the difference between the INS position and the CNS position, and INS velocity and the DVL velocity, which is:

(28) $$Z_{M1}={\left[\begin{array}{cccc} \varphi -\varphi_{CNS} & \lambda -\lambda_{CNS} & v_{x}-v_{xDVL} & v_{y}-v_{yDVL} \end{array}\right]}^{T}$$

The initial value of the centralized Kalman Filter should be set before navigation: 

**Q**_*M*1_ is the covariance matrix of the system noise, and the standard of setting the initial value of **Q**_*M*1_ is the IMU error. The initial value of **Q**_*M*1_ is:

(29) $$Q_{M1}(0)=diag\left({\left[\begin{array}{lllll} 5\times 10^{-5} & 5\times 10^{-5} & 8.73\times 10^{-7} & 8.73\times 10^{-7} & 8.73\times 10^{-7} \end{array}\right]}^{2}\right)$$

**P**_*M*1_ is the mean square error matrix. According the principle of Kalman Filter, the initial value of **P**_*M*1_ can be set big enough in order to ensure the estimation accuracy, therefore:

(30) $$\begin{array}{l} P_{M1}(0)=diag([\begin{array}{lllllll} 0.1 & 0.1 & 1.6205\times 10^{-6} & 1.6205\times 10^{-6} & 1.7453\times 10^{-4} & 1.7453\times 10^{-4} & 1.7453\times 10^{-4} \end{array}\\ {\begin{array}{llllllll} 4.8481\times 10^{-8} & 4.8481\times 10^{-8} & 4.8481\times 10^{-8} & 1.0\times 10^{-4} & 1.0\times 10^{-4} & 1.0\times 10^{-4} & 0.1 & 0.1 \end{array}]}^{2}) \end{array}$$

**R**_*M*1_ is the covariance matrix of system measurement noise. It is assumed that the measurement noise of CNS is Gaussian white noise with amplitude of 1 meter, and the measurement noise of DVL is color noise. Hence, the initial value of **R**_*M*1_ is:

(31) RM1(0)=diag([1/Re1/Re0.010.01]2)

The measurement covariance during the navigation process is adjusted adaptively as follows, and the adjusting process is suitable for Method 2 and Method 3:

(32) RM1(t)=diag([y(t)/Rey(t)/Re0.010.01]2)

The method of generating color noise is arbitrarily, the method used in this paper is shown as follows:

(33) $$e(k)=\frac{0.2+0.1z^{-1}+0.04z^{-2}}{1-1.5z^{-1}+0.7z^{-2}+0.1z^{-3}}\xi (k)$$

where *ξ*(*k*) is the Gaussian white noise with amplitude of 1. *e*(*k*) is the generating color noise.

*Method 2*:

The Federated Filter is introduced in the method 2, and the difference between method 2 and the method proposed in this paper is that there is no adaptive parameter in method 2. The information sharing factor in method 2 is constant, these are *β*_1_ = *β*_2_ = 0.5 and *β_m_* = 0. The initial values of the Federated Filter can refer to the setting value in the centralized Kalman Filter, and they are:

$$Q_{1}(0)=Q_{2}(0)=Q_{m}(0)=Q_{M1}(0),\;P_{1}(0)=P_{2}(0)=P_{m}(0)=P_{M1}(0),$$

(34) R1(0)=diag([1/Re1/Re]2), R2(0)=diag([0.010.01]2)

where subscripts 1 and 2 are the parameters setting for local filter 1 and 2 respectively, and subscript m is the parameters setting for main filter.

*Method 3*:

Method 3 is the integrated navigation method using AISFF, which is described in Section 4. The parameters setting are same with that in method 2. The sampling frequency of the sensors in simulation is 1 Hz, and the observation time of the measurement in simulation is setting with 20 s. Therefore, the window size M in Equation (9) is 20 (20 = 1 Hz × 20 s).

*Method 4*:

Neuro-Fuzzy network is introduced in Method 4. For this method, a Neural Network is a multilayer feed forward neural network. Transmitting signal forward and error backward is the main feature. In the process of signal forward passing, the input signal, which is introduced from the input layer, is transmitted by the hidden layer, and then output by the output layer. When the output signal is not the desired value, the transmission direction will change over to back propagation. And the forecast errors can be obtained.

#### 5.1.2. Simulation Results

**Figure 11:**
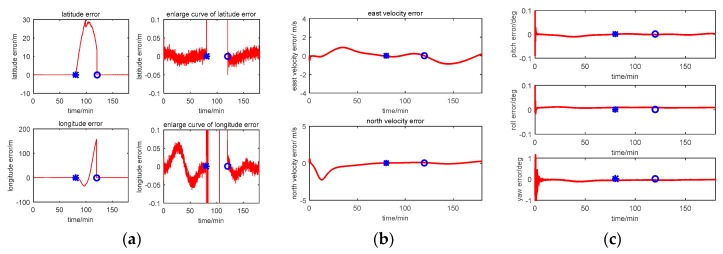
Simulation result with Method 1 (centralized Kalman Filter). (**a**) Position error; (**b**) Velocity error; (**c**) Attitude error.

**Figure 12:**
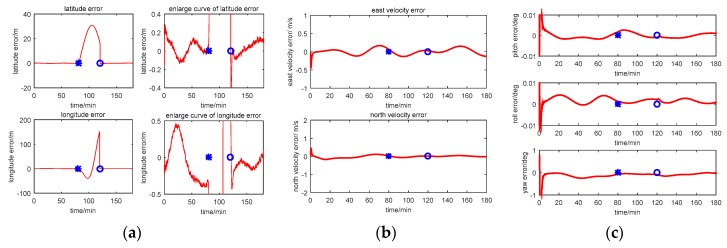
Simulation result with Method 2 (Federated Filter). (**a**) Position error; (**b**) Velocity error; (**c**) Attitude error.

**Figure 13:**
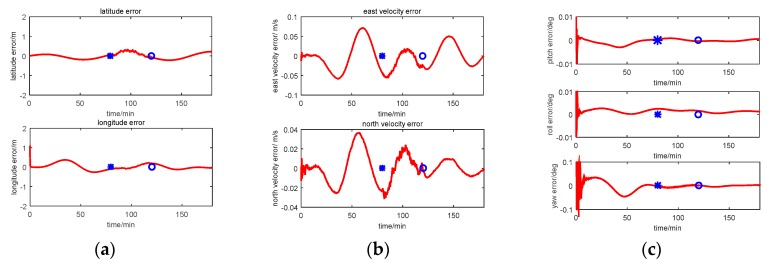
Simulation result with Method 3 (AISFF). (**a**) Position error; (**b**) Velocity error; (**c**) Attitude error.

**Figure 14:**
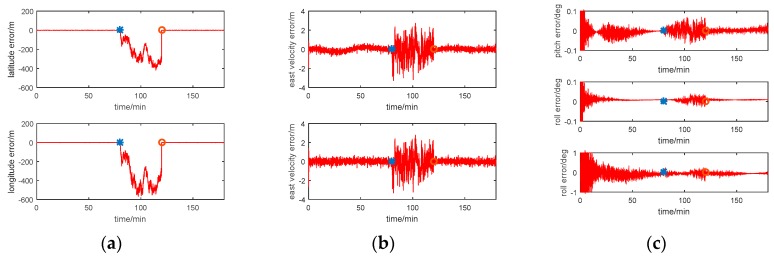
Simulation result with Method 4 (NF). (**a**) Position error; (**b**) Velocity error; (**c**) Attitude error.

**Figure 15:**
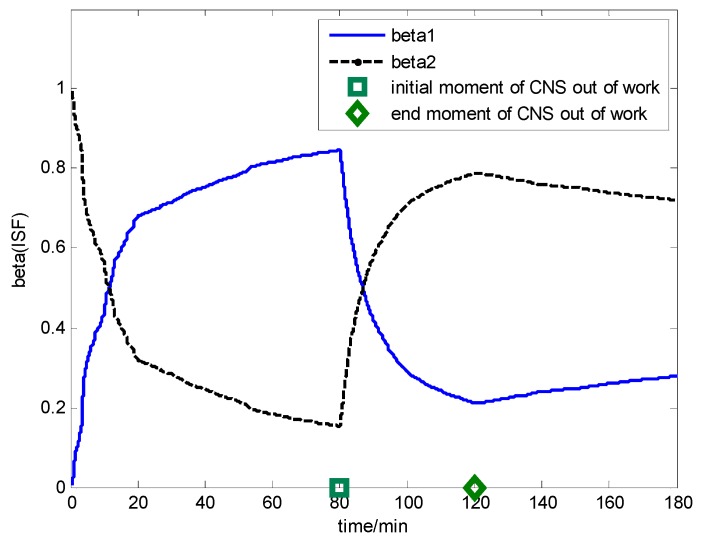
Simulation result for ISF.

**Figure 16:**
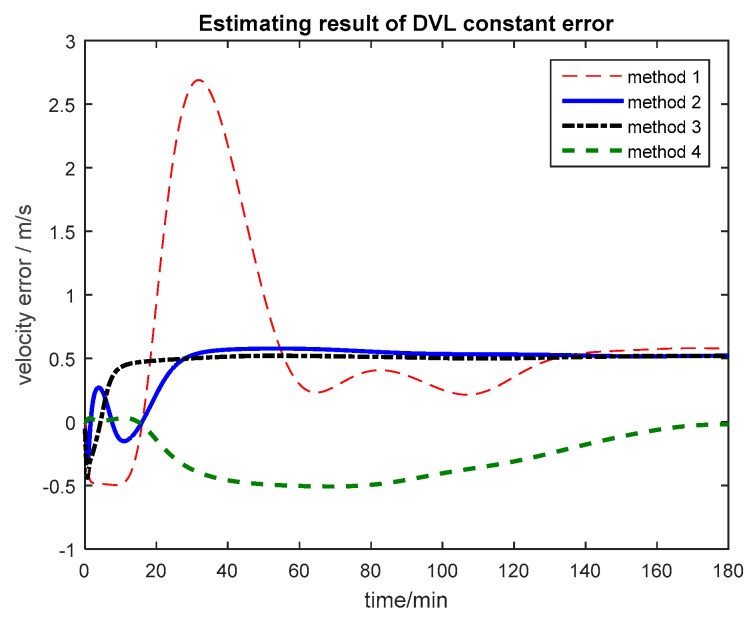
Estimating result of DVL constant error.

The multi-sensor integrated navigation results of INS/CNS/DVL are shown in Figure 11, Figure 12, Figure 13 and Figure 14. The initial moment and the end moment of outage of CNS are labeled on the x axis (time axis) in each curve. Figure 15 is the trend of ISF, and Figure 16 is the estimation result of DVL constant velocity error.

Comparing the position error in figures (a) of Figure 11, Figure 12 and Figure 13, there are some difference between the estimation results based on the three methods when the CNS out of work during the time period of 80~120 min. According to the simulation results with Method 1, it can be seen that when the CNS out of work, the estimation results of the position error is diverged rapidly. However, based on adjusting the measurement covariance for the CNS accordingly, the estimation position error is much less than the CNS position error (position error with 500 m according to the simulation conditions). Hence, the influence of the CNS position error on the estimation results can be decreased by adjusting the measurement covariance for CNS. Because ISF of Method 2 is setting with constants of 0.5, the results are the same with that of Method 1. Comparing the position error from Method 2 with that from Method 3, it can be seen that the accuracy of the navigation error of Method 3 is much better than that of Method 2. During the time period of 0~80 min, when the CNS is operating smoothly, both the ISF of two subfilters are set at 0.5, hence, the contribution of position error is equal to that of the velocity error, and the influence of the DVL measurement color noise on the estimation result is not adjusted. The influence of the DVL measurement color noise on the estimation result is adjusted by AISFF. It can be observed more clearly by latitude error and longitude error, the position error calculated by Method 2 increases rapidly when the CNS is not working, and it converges after 120 min. However, the position error calculated by Method 3 is stable when the CNS is not working, which is caused by increasing the contribution of subfilter 2 to the main filter by ISF. Therefore, the fault-tolerance performance of federated filter is improved by introducing ISF in the filter.

According to the estimation results of the velocity error with three methods, the estimation accuracy and stability of Method 1 is similar to that of Method 2. It is because not all of the influence of color noise in DVL on the estimation result can be avoided. Comparing the velocity error in Method 3 with that in the other two methods, the accuracy of estimating the velocity error is better than that of the other two methods. It is because of the ISF, the contribution of subfilter 2 to the main filter is decreased due to the DVL color noise, and for the subfilter 1, when the observation of Kalman Filter is the position error, and the velocity error can be estimated. 

For the estimation results of the attitude error with the first three methods, the estimation accuracy and stability of Method 1 is similar to those of the other two methods. It is because when the observation of Kalman Filter is the position error or the velocity error, the attitude error can be estimated. Therefore, when the CNS is out of work, the contribution of subfilter 2 is increased by ISF, and the accuracy of attitude cannot be influenced.

The estimation result for the Method 4 is worse than that of the other three methods. Especially for the time period of 80–120 min, the integrated navigation errors are not estimated and compensated by the Neuro Fuzzy method. Therefore, the fuzzy system can be used for the signal outages for a short time.

According to the ISF, it is obtained that the accuracy of the position information from CNS is better than that of DVL in the initial state (0~80 min). Hence, the contribution of subfilter 1 should be more than that of subfilter 2, which is the reason of beta1 increasing and beta2 decreasing in Figure 15. When the CNS is out of order during the time period of 80~120 min, the accuracy of the position information from CNS is worse than that of DVL, hence, the contribution of subfilter 1 should be less than that of subfilter 2, which is shown in the time period of 80~120 min (beta1 decreasing and beta2 increasing). When CNS is recovered and working normally, it is seen that the contribution of subfilter 1 is more than that of subfilter 2, which is shown in the time period of 120~180 min (beta1 increasing and beta2 decreasing). 

Based on the curves in Figure 16, the DVL constant error can be estimated by introducing this item as the state vector by the first three methods, but the time to converge of the algorithm based on the Federated Filter is less than that of Kalman Filter and Neuro-Fuzzy method. The influence of DVL constant error on the integrated navigation system can be avoided.

### 5.2. Experiments and Results

#### 5.2.1. Experiment Conditions

In this section, experiments are carried on the lake to further verify the superiority of the integrated navigation method for INS/CNS/DVL using AISFF. The INS, CNS and DVL are installed on the ship. The INS is developed by our lab. The main sensors of INS are gyros and accelerometers, and measurement constant error of theses sensors (including gyro constant drift, gyro scale factor and installation error, accelerometer bias, accelerometer scale factor and installation error) are estimated and compensated by the equipment test in the laboratory. For the testing methods readers can refer to [54]. In addition, the gyro drift and the accelerometer bias in Equation (2) are the estimation error, which is the difference between the IMU real output error and the estimation result in the laboratory. The performance of the gyros and accelerometers are shown in Table 2.

The depth of the lake is about 100 m, so the DVL velocity can be obtained during all the experiment process. The installation error between DVL and INS is measured and compensated before the integrated navigation experiment. Hence, the influence of the installation error on the integrated navigation system can be avoided. The star sensor is used as the sensor of CNS. The performance is shown in Table 2. As the reference information, which is used as the judging standard of the integrated navigation information accuracy and calculating position error from that, GPS is introduced in the experiment with the accuracy is 10 m, and comparing the position from the integrated system with GPS position is the way to judge the integrated accuracy. Laptops are used to gather acquisition data in this experiment, to compare and analyze the effects and precision of the integrated algorithm.

**Figure 17:**
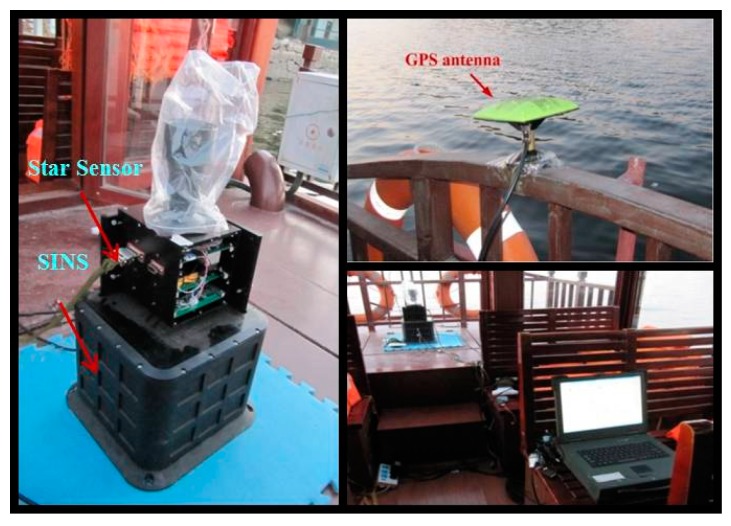
The experimental devices.

**Figure 18:**
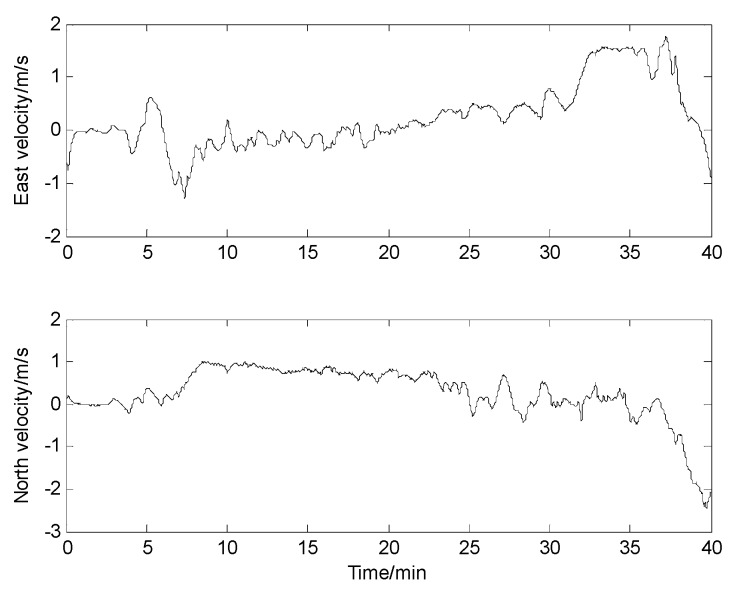
Speed of the ship during the experiment.

The experimental devices are shown in Figure 17. The speed of the ship along east and north of the navigation frame during the experiment is shown in Figure 18. And based on the speed of the ship, it can be concluded that the total path length for the experiment is about 1945 m. 

According to the performance index in Table 2 and the design principle, the initial value of the matrixes for the filters can be set as follows:

*Method 1*:

(35) $$Q_{M1}(0)=diag\left({\left[\begin{array}{lllll} 9.85\times 10^{-3} & 9.85\times 10^{-3} & 2.42\times 10^{-7} & 2.42\times 10^{-7} & 2.42\times 10^{-7} \end{array}\right]}^{2}\right)$$

(36) $$\begin{array}{l} P_{M1}(0)=diag([\begin{array}{lllllll} 0.1 & 0.1 & 1.6205\times 10^{-5} & 1.6205\times 10^{-5} & 8.726\times 10^{-3} & 8.726\times 10^{-3} & 8.726\times 10^{-3} \end{array}\\ {\begin{array}{llllllll} 4.93\times 10^{-3} & 4.93\times 10^{-3} & 4.93\times 10^{-3} & 1.0\times 10^{-4} & 1.0\times 10^{-4} & 1.0\times 10^{-4} & 0.1 & 0.1 \end{array}]}^{2}) \end{array}$$

(37) $$R_{M1}(0)=diag\left({\left[\begin{array}{cccc} 7.83\times 10^{-5} & 7.83\times 10^{-5} & 0.1 & 0.1 \end{array}\right]}^{2}\right)$$

*Method 2*:

(38) $$Q_{1}(0)=Q_{2}(0)=Q_{m}(0)=Q_{M1}(0)$$

(39) $$P_{1}(0)=P_{2}(0)=P_{m}(0)=P_{M1}(0)$$

(40) $$R_{1}(0)=diag\left({\left[\begin{array}{cc} 7.83\times 10^{-5} & 7.83\times 10^{-5} \end{array}\right]}^{2}\right),R_{2}(0)=diag\left({\left[\begin{array}{cc} 0.1 & 0.1 \end{array}\right]}^{2}\right)$$

*Method 3*:

The parameters setting are same with that of Method 2. Before the navigation, the process of INS coarse alignment is carried out to estimate the body’s initial attitude [55,56].

*Method 4*:

Similar with simulation, the Neuro-Fuzzy network is introduced in Method 4. For the parameter settings readers can refer to [13,19].

#### 5.2.2. Experiment Results

**Figure 19:**
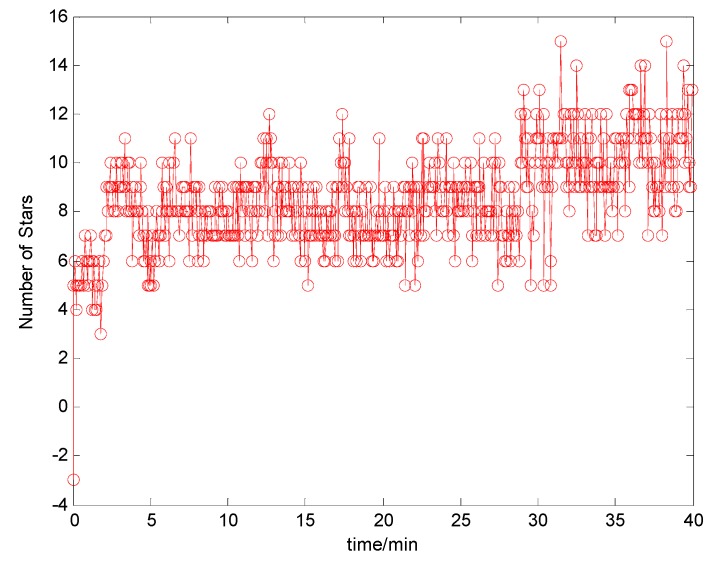
Numbers of Stars by CNS during experiment.

**Figure 20:**
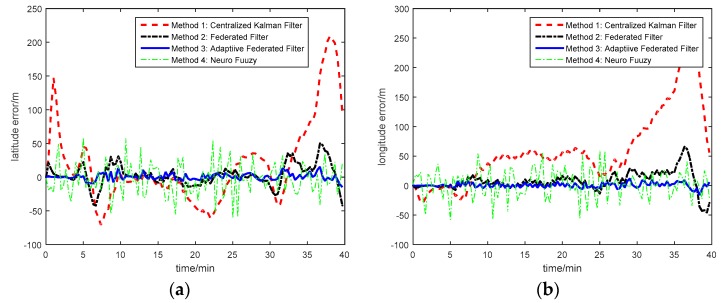
Experiment result with three integrated navigation methods: (**a**) Latitude error; (**b**) Longitude error.

Figure 19 is the star number during the experiment. The analysis of the position accuracy (latitude error and longitude error) by these four methods is described in Figure 20, and the latitude error and longitude error are the difference between the calculated position and the GPS position. 

According to the experimental results from Figure 20, it can be obtained that, at the beginning of the experiment, the latitude error calculated by Method 1 diverges and converges quickly. It is because the number of stars by CNS is less, so the accuracy of position from CNS is decreased. However, the contribution of CNS position and DVL velocity to the main filter is separated by Methods 2 and 3. Hence, the influence of CNS position on the estimation result at the beginning by Methods 2 and 3 are less than that by Method 1. However, the stability of the position error calculated by the Neuro-Fuzzy method is not well. Compared with the centralized Kalman Filter, the Federated Filter with constant ISF and the Neuro-Fuzzy method, the vehicle’s position can be estimated and tracked better by the multi-sensor integrated navigation system of INS/CNS/DVL using AISFF. This implies that the INS error can be estimated and compensated by introducing the CNS and DVL as the reference system using AISFF, and the influence of the DVL measurement error and the outage of CNS on the integrated navigation system can be avoided, and the accuracy of the integrated navigation information is improved.

## 6. Conclusions

According to the problem in the USV’s navigation mode without the satellite navigation system, the multi-sensor integrated navigation method of INS/CNS/DVL using AISFF is proposed in this paper. Based on the principle of INS, CNS and DVL, the challenges of introducing the DVL velocity and the CNS position are discussed. Therefore, the premises of using CNS and DVL as the reference systems to correct the INS navigation error are estimate and correct the DVL measurement error, and ensure the stability of the INS/CNS/DVL integrated navigation system when the CNS cannot work under bad weather conditions. In order to resolve the problem, the ASIFF is introduced as the data fusion method: firstly, the DVL constant error is used as the state variable. Thus, this constant error can be estimated and compensated, and the influence of the DVL constant error on the integrated navigation accuracy can be avoided; secondly, the information sharing factor of the Federated Filter is adaptive adjusted to improve overall system reliability by maintaining multiple component solutions usable as back-ups. The influence of the DVL measurement noise and the CNS outages on the integrated navigation system can be avoided. Furthermore, this integrated navigation result is verified by simulation, which shows that the INS/CNS/DVL integrated navigation method based on AISFF can effectively estimate and compensate the INS navigation error. In the end, a lake test verified the validity and engineering applicability of the method.

## Figures and Tables

**Table 1:** Vehicle’s trajectory of the simulation.

No.	State	Value	Duration
1	Moving forward with constant speed	5 m/s	30 min
2	Acceleration motion with constant	0.1 m/s^2^	10 s
3	Moving forward with constant speed	6 m/s	20 min
4	Left turning	1°/s	90 s
5	Moving forward with constant speed	6 m/s	10 min
6	Right turning	1°/s	90 s
7	Moving forward with constant speed	6 m/s	20 min
8	Left turning	2°/s	45 s
9	Moving forward with constant speed	6 m/s	10 min
10	Right turning	1°/s	45 s
11	Moving forward with constant speed	6 m/s	10 min
12	Decelerated motion with constant	0.1 m/s^2^	10 s
13	Moving forward with constant speed	5 m/s	20 min

**Table 2:** The Gyro and the star sensor performance index.

Parameter Item		Index
Gyro	Dynamic Range	±100 °/s
	Bias Stability	≤0.05 °/h
	Random Walk	≤0.005 °/$\sqrt{h}$
	Nonlinear Degree of Scale Factor	≤20 ppm
Accelerometers	Bias Stability	100 μg
	Nonlinear Degree of Scale Factor	≤20 ppm
Star Sensor	Field of view	24°
	Level of star observation	no less than +7 level
	Data update rate	20 Hz
	Attitude accuracy	5″
	Dynamic Range	20°/s
